# Elevated Baseline Neutrophil Count Correlates with Worse Outcomes in Patients with Muscle-Invasive Bladder Cancer Treated with Chemoradiation

**DOI:** 10.3390/cancers15061886

**Published:** 2023-03-21

**Authors:** Sébastien Meunier, Alexandre Frontczak, Loïc Balssa, Julie Blanc, Salim Benhmida, Mandy Pernot, Magali Quivrin, Etienne Martin, Yasser Hammoud, Gilles Créhange, Jihane Boustani

**Affiliations:** 1 Department of Radiation Oncology, Centre Georges François Leclerc, 21000 Dijon, France; 2 Department of Urology, University Hospital of Besançon, 25000 Besançon, France; 3 Department of Biostatistics, Centre Georges François Leclerc, 21000 Dijon, France; 4 Department of Radiation Oncology, University Hospital of Besançon, 25000 Besançon, France; 5 Department of Radiation Oncology, Institut Curie, 92210 Saint-Cloud, France; 6 INSERM, EFS BFC, UMR1098, RIGHT, Interactions Greffon-Hôte-Tumeur/Ingénierie Cellulaire et Génique, University of Bourgogne Franche-Comté, 25000 Besançon, France

**Keywords:** neutrophil-to-lymphocyte ratio, neutrophil count, localized bladder cancer, chemoradiation, inflammation

## Abstract

**Simple Summary:**

Inflammation plays a role in the development and prognosis of bladder cancer. We aimed at studying the prognostic significance of neutrophil-to-lymphocyte ratio (NLR) and neutrophil count (PNN) at baseline in patients with localized bladder cancer treated with chemoradiation. High NLR > 2.6 was associated with shorter overall survival (OS) in univariate analysis only, whereas high PNN > 4000/mm^3^ was associated with shorter OS and progression-free survival in univariate and multivariate analyses. Along with other established prognostic factors, baseline PNN could serve as a biomarker to incorporate in a novel nomogram for selecting patients who might benefit from a bladder preservation strategy.

**Abstract:**

Background: The role of inflammation in the development and prognosis of bladder cancer (BC) is now established. We evaluated the significance of neutrophil-to-lymphocyte ratio (NLR) and neutrophil count (PNN) in patients with localized BC treated with chemoradiation. Methods: Clinical characteristics and baseline biological data were retrospectively collected. We tested the association between NLR, PNN, and overall survival (OS) and progression-free survival (PFS). Results: One hundred and ninety-four patients were included. Median PNN was 4000.0/mm^3^ [1500.0–16,858.0] and median NLR was 2.6 [0.6–19.2]. In patients with NLR > 2.6, median OS and PFS were lower (OS: 25.5 vs. 58.4 months, *p* = 0.02; PFS: 14.1 vs. 26.7 months, *p* = 0.07). Patients with PNN > 4000/mm^3^ had significantly lower OS (21.8 vs. 70.1 months, *p* < 0.001) and PFS (13.7 vs. 38.8 months, *p* < 0.001). Contrary to NLR, PNN > 4000/mm^3^ was associated with shorter OS and PFS in multivariate analysis. Conclusions: Elevated PNN at baseline was associated with worse OS and PFS. NLR was not an independent prognostic factor.

## 1. Introduction

Bladder cancer ranks ninth among all cancers worldwide in terms of incidence with 430,000 new cases diagnosed in 2012, and 13th worldwide in terms of mortality with 165,000 deaths in 2012 [1]. Forty-five percent of bladder cancers are diagnosed after the age of 75 years [2]. Currently, the standard treatment for non-metastatic MIBC is neoadjuvant chemotherapy (CT) followed by radical cystectomy with extended lymph node dissection [3]. Cisplatin-based neoadjuvant CT was shown to improve survival outcomes with an 8% absolute improvement in 5-year overall survival (OS) [3]. However, this treatment leads to a high morbidity (30%), a perioperative mortality of 2–3%, and a decreased quality of life with urinary and gastrointestinal toxicities [4]. In a population that is generally aged over 70 years with frequent comorbidities, surgery is often contra-indicated or rejected by the patients. A multimodal treatment (MMT) comprising transurethral resection of the bladder tumor (TURBT) followed by chemoradiation (CRT) is an alternative in selected, well-informed, and compliant patients. Several studies have reported equivalent outcomes between MMT and RC [5,6] but no randomized trial has compared these two strategies to date. Several prognostic and predictive factors are well established in MIBC, such as advanced T and N stage, tumor size 3–5 cm, hydronephrosis, multifocality, incomplete TURBT, age (>70 years old), association with carcinoma in situ (CIS), and renal failure [7]. Over the last decade, several studies have shown the role of inflammation in the development, progression, and metastatic evolution of cancers [8], inducing proliferation, survival, and migration in the tumor micro-environment [9]. During systemic inflammation, polynuclear neutrophils (PNN) increase, whereas lymphocytes (Lc) decrease, leading to a modification of the neutrophil-to-lymphocyte ratio (NLR) defined as PNN/Lc [10]. Several meta-analyses have suggested the role of the NLR as a prognostic marker in recurrence, metastatic progression, and survival of bladder cancer [11,12,13]. The influence of PNN on the development of cancers has also been demonstrated in promoting tumor proliferation, angiogenesis, tumor cell migration, and metastasis [14].The objective of this retrospective study was to test the association between NLR, PNN, and clinical outcomes in patients with MIBC treated by CRT.

## 2. Materials and Methods

### 2.1. Study Population

We identified in the informatics database of each hospital all patients treated by CRT with curative intent for a non-metastatic MIBC between April 1996 and March 2019 at the Georges Francois Leclerc Center in Dijon and at the University Hospital of Besançon, France. CRT was offered if there were medical and/or surgical contra-indications to cystectomy, or when patients declined surgery. Patients were excluded if they had exclusive radiotherapy (RT) without CT, palliative RT, and/or metastatic disease. Monitoring after treatment consisted in clinical assessment at the end of CRT and follow-up between the urologist and radiotherapist every 3 to 6 months. Cystoscopy was performed every 3–6 months for at least 5 years, and then every year for life. A thoracic-abdominal and pelvic computed tomography scan was performed at least once a year.

### 2.2. Covariates and Outcomes

Data were retrospectively collected from the patients’ clinical records. The following characteristics were identified: age at diagnosis, Charlson comorbidity index, T and N stage, histological type, tumor size (> or ≤5 cm), hydronephrosis, association with CIS, uni- or multifocal tumor, complete or incomplete TURBT, neoadjuvant CT, kidney failure, RT dose to the pelvis and bladder, fractionation, treatment interruption, RT technique, type of CT, number of CT cycles, compliance with CT, baseline leucocytes, PNN, Lc, and NLR (PNN/Lc). Baseline blood parameters were performed up to seven days before the beginning of the treatment.

Acute toxicity was defined as hematological, renal, cardiac, urinary, or digestive adverse effects attributable to the treatment and occurring from the first day of RT to 3 months after the end of irradiation. Late toxicity was defined as urinary or digestive adverse effects attributable to the treatment lasting or occurring more than 3 months after the end of RT.

Complete response was defined as the absence of local and distant recurrence during monitoring. Evaluation of clinical response was based on the RECIST criteria version 1.1. Local recurrence was defined as visible tumor on cystoscopy or positive tumor site biopsy. Metastatic recurrence was defined as the detection of one or more metastases on tomography scan. OS was defined as the time from treatment initiation to death from any cause. Surviving patients were censored at the date of last follow-up. Progression-free survival (PFS) was defined as the time from treatment initiation to disease progression or death from any cause. Surviving patients without disease progression were censored at the date of last follow-up. Acute and late toxicities were evaluated using the Common Terminology Criteria for Adverse Events (CTCAE) criteria, version 5.0. This study was approved by the local Institutional Review Board.

### 2.3. Statistical Analysis

The objective of this study was to test the association between NLR, PNN, and survival outcomes. The median NLR and PNN were used as cut-offs to define low and high NLR and PNN groups, respectively. Univariate analyses were carried out to compare the clinical, biological, and pathological characteristics between the two groups of patients according to NLR and PNN. For this comparison, the Chi2 test or Fischer’s exact test were used for the categorical variables and the Student *t* test or the Wilcoxon test (depending on the normality of the distribution) were used for the quantitative variables. Univariate and multivariate logistic regression analyses were performed to test the impact of NLR and PNN on local recurrence and metastatic recurrence. Kaplan–Meier plots graphically depicted univariable survival rates in the overall population and according to NLR and PNN. The statistical significance of differences among NLR groups and PNN groups was tested with the log-rank test. Multivariate Cox regression models were used to assess the impact of NLR and PNN, among other confounding factors, on OS and PFS. These factors were determined according to the criteria published in the literature known to influence survival outcomes. A *p*-value < 0.05 was considered statistically significant. All analyses were performed using SAS software version 9.4.

## 3. Results

### 3.1. Patients’ Characteristics

Between 1996 and 2019, 194 patients with T2–T4 N0–N3 M0 MIBC received CRT. The patients’ characteristics are displayed in Table 1. Median age at diagnosis was 79.0 years [55.0–94.0] and median follow-up was 37.5 months [1.0–213.5]. Patients were predominantly men (75%) with a Charlson score ≥ 5 in 91% patients. The main histological type was urothelial carcinoma (94%), and CIS was present in 23 (12%) cases. At baseline, median PNN count was 4000.0/mm^3^ [1500.0–16858.0], median Lc count was 1625.0/mm^3^ [190.0–3700.0], and median NLR was 2.6 [0.6–19.2]. After TURBT, which was complete in 107 patients (72%), RT was delivered to the pelvic lymph nodes and to the whole bladder (Supplementary Table S1). Pelvic RT was performed in 180 patients (93%) at a median dose of 45Gy [45.0–60.0]. The median dose to the bladder was 64.8Gy [50.0–70.0]. Hypofractionated RT was performed in 13 patients (7%) (50Gy in 20 fractions (*n* = 1); 55Gy in 20 fractions (*n* = 12)). Overall, RT was interrupted in 21 patients (11%): in 15 patients because of an intercurrent event (72%) defined as an event unrelated to toxicity or progression leading to treatment interruption; in three patients due to a treatment-related toxicity (14%), and in three patients due to on-treatment progression (14%). CT was interrupted in 51 patients (26%) because of toxicity, intercurrent event, or treatment progression. Acute and late toxicities are shown in Supplementary Table S2. Acute toxicities were observed in 84% of the patients, predominantly grade 1. Acute grade 2 or higher toxicities were observed in 24% patients, without any grade 4 or 5 adverse event. The most frequent acute toxicities were urinary and gastrointestinal. Late grade 1–2 toxicities were seen in 37% patients without any grade 3+ event.

Table 2 shows the comparison of patients’ characteristics according to baseline NLR and PNN. This analysis was performed in 178 patients with available biological data at baseline. There was significantly more hydronephrosis in the high NLR (>2.6) group (40% vs. 25%, *p* = 0.04). There were significantly more men (80% vs. 67%, *p* = 0.04), incomplete TURBT (39% vs. 20%, *p* = 0.02), kidney failure (50.5% vs. 31%, *p* = 0.01), and local recurrence (24% vs. 9%, *p* = 0.01) in the high PNN (>4000/mm^3^) group. There was no significant difference in distant recurrence and toxicities according to baseline NLR and PNN.

### 3.2. Association between NLR, PNN, and Outcomes

At first evaluation, 130 patients (67%) had complete response, four (2%) had partial response, and nine (4.6%) had stable disease. During follow-up, local recurrence occurred in 25 patients (13%) and metastatic recurrence in 59 patients (30%). The most common metastatic sites were the lung (33%), extra-pelvic nodes (18%), bone (17%), and liver (14%). Other sites (peritoneal, pleural, adrenal, and brain) were seen in 13% and pelvic lymph node in 5%. Salvage cystectomy was performed in three (1.6%) patients because of local recurrence.

**Figure 1:**
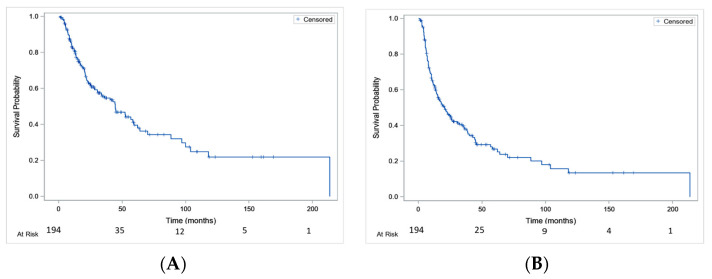
Overall survival (**A**) and progression-free survival (**B**) in the whole studied population.

Median OS was 44.5 [30.7–59.1] months, with a 1-year OS rate of 82% [75.1–86.6%] and a 4-year OS rate of 47% [37.9–55.4%] (Figure 1A). Median PFS was 20.4 months [14.5–26.7] with a 1-year PFS rate of 62.8% [55.3–69.3%] and a 4-year PFS rate of 29.4% [21.9–37.2%] (Figure 1B).

**Figure 2:**
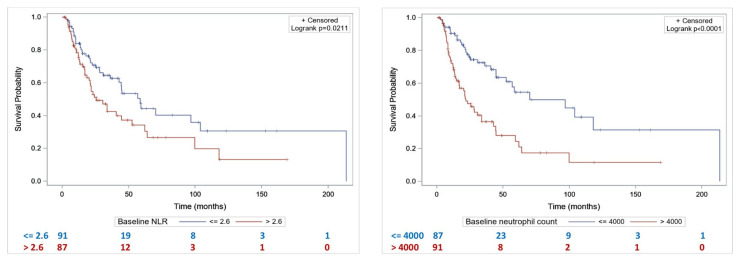
Overall survival according to NLR (**A**) and baseline neutrophil count (**B**).

Median OS was significantly lower in patients with baseline NLR > 2.6 compared to patients with NLR ≤ 2.6 (25.5 months [20.2–52.4] vs. 58.4 months [42.7–103.9], *p* = 0.02) (Figure 2A). Median OS was significantly lower in patients with baseline PNN > 4000/mm^3^ compared to patients with PNN ≤ 4000/mm^3^ (21.8 months [14.9–33.7] vs. 70.1 months [44.7–], *p* < 0.001) (Figure 2B).

By univariate analysis, T3–T4 stage, hydronephrosis, baseline PNN > 4000/mm^3^), and baseline NLR > 2.6 were significantly associated with shorter OS (Table 3). By multivariate analysis, baseline PNN > 4000/mm^3^ and T3–T4 stage were significantly associated with shorter OS (Table 3).

**Figure 3:**
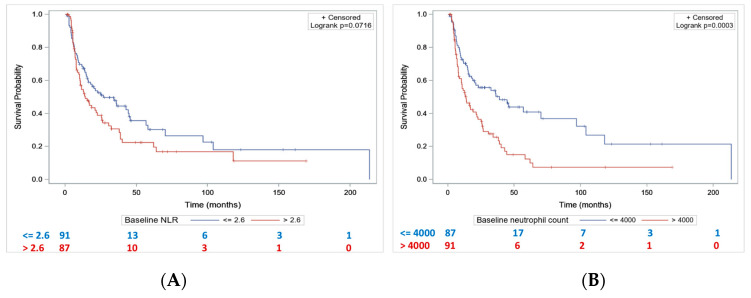
Progression-free survival according to baseline NLR (**A**) and baseline neutrophil count (**B**).

Median PFS was lower in patients with baseline NLR > 2.6 compared to patients with NLR ≤ 2.6 (14.1 months [10.2–22.6] vs. 26.7 months [15.9–44.7], *p* = 0.07) (Figure 3A). Patients with baseline neutrophil count > 4000/mm^3^ had a significantly lower median PFS compared to patients with baseline PNN ≤ 4000 (13.7 months [8.5–21.4] vs. 38.8 months [17.9–96.8], *p* = 0.0003) (Figure 3B).

By univariate analysis, T3–T4 stage, hydronephrosis, and baseline PNN > 4000/mm^3^ were significantly associated with shorter PFS (Table 4). By multivariate analysis, only baseline PNN > 4000/mm^3^ was significantly associated with shorter PFS (Table 4).

## 4. Discussion

Systemic inflammation is a recognized characteristic of malignancy, and several inflammatory markers have been investigated as prognostic indicators for cancer patients. For instance, elevation of C-reactive protein before treatment predicted a poor prognosis in patients with MIBC [15]. In our study, we focused on NLR and PNN and showed that baseline NLR was not associated with OS or PFS as opposed to several studies showing an association between NLR and survival [11,12,16,17,18,19,20,21]. A pooled analysis of 17 studies and 11,262 patients treated for bladder cancer with MMT or radical cystectomy showed a significant reduction in OS (HR = 1.27, 95% CI = 1.12–1.43), PFS (HR = 1.75, 95% CI = 1.36–2.15), and cancer-specific survival (HR = 1.27, 95% CI = 1.19–1.35) in patients with an elevated NLR [16]. However, the NLR ranged between 2 and 5. Additionally, studies with neutral results were not included in this meta-analysis due to insufficient data, which may have contributed to publication bias. Moreover, some studies reported only univariate results, which may have overestimated the prognostic role of NLR. Our results are in line with a secondary analysis of a phase III trial, SWOG 8710, that assessed radical cystectomy with or without neoadjuvant CT in 317 patients with MIBC [22]. This was the first analysis of NLR in MIBC to use prospectively collected clinical data. In contrast to previous studies, NLR was neither a prognostic nor a predictive biomarker for OS after 18-year follow-up. Therefore, we suggest that NLR is not directly linked to cancer prognosis, but reflects the inflammation related to local factors. Indeed, in our study, patients with a high baseline NLR had significantly more hydronephrosis than patients with low NLR. Hydronephrosis is known to increase intracavity pressure and induce synthesis of prostaglandin E2 by cyclooxygenases [23]. Prostaglandin E2 lead to an increase in vascular permeability, recruitment, and activation of PNN [23,24,25,26]. Thus, NLR might be influenced by hydronephrosis that leads to inflammation. A correlation study between hydronephrosis and NLR would be of interest to prove this hypothesis. Another explanation for our results is the fact that NLR can be increased in conditions other than cancer, such as smoking, diabetes, and chronic inflammatory diseases [12]. However, we did not study the correlation between these factors and NLR in our cohort.

On the other hand, baseline PNN was associated with OS and PFS in multivariate analyses. To our knowledge, this is the first report of the association of PNN with survival outcomes in localized MIBC. A similar association has been described in the metastatic setting in patients with renal cell carcinoma [27] and melanoma [28]. It has also been shown in localized endometrial cancer [29] and in advanced gastric cancer [30]. The main hypothesis of the association between high PNN count and poor clinical outcomes is the involvement of inflammation in the initiation, progression, and metastatic course of cancer [12]. Inflammation increases vascular permeability, infiltration into lymphatic and blood vessels, adhesion to the endothelium, and metastatic migration [12]. Neutrophils are the first line of defense during inflammation [14]. Tumors seem to induce neutrophilia by producing neutrophil-attracting chemokines such as interleukin (IL)-8 [14]. In addition, neutrophils activate a positive feedback mechanism by releasing chemokines that attract more neutrophils into the tumor. Several mechanisms are involved in the protumoral activity of neutrophils. Tumor-associated neutrophils promote angiogenesis, chronic inflammation, and immunosuppression [14]. They can also induce migration of tumor cells and promote tumor cell invasion [31]. Moreover, neutrophils seem to facilitate metastases creating a permissive growth environment before the arrival of tumor cells in premetastatic niches [32]. Since patients with high PNN had worse survival outcomes, they might not be ideal candidates for bladder preservation strategies as hinted by the higher local recurrence rate in the high PNN group.

Optimal candidates for bladder preservation with CRT include patients with unifocal T2–T3 tumors that are <6 cm, without hydronephrosis, and without extensive CIS [33]. In our study, higher clinical T stage (T3/T4 vs. T2), hydronephrosis, and incomplete TURBT were associated with decreased OS. These associations have been observed in other large series [34,35,36,37]. Other factors such as advanced age, tumor multifocality, lymph node involvement, and extensive CIS are also associated with a higher risk of recurrence and/or decreased survival [38].

The wide range of prognoses after cystectomy has led to the development of several post-surgical prognostic nomograms such as the International Bladder Cancer Nomogram Consortium (IBCNC) nomogram and the Bladder Cancer Research Consortium (BCRC) [39]. In locally advanced and metastatic MIBC, Yang et al. established a neutrophil-based prognostic model incorporating five neutrophil-related genes (EMR3, VNN1, FCGRT, HIST1H2BC, and MX1) [40]. FCGRT was identified as the key neutrophil-related gene linked to an adverse prognosis of bladder cancer. Upregulation of FCGRT indicated activated cancer metabolism, immunosuppressive tumor environment, and dysregulated functional status of immune cells. FCGRT overexpression was also correlated with decreased expression of PD-L1 and low levels of tumor mutation burden. FCGRT predicted a poor response to immunotherapy and had a close correlation with chemotherapy sensitivity. In bladder preservation strategies, it would be interesting to construct a novel nomogram based on the abovementioned established prognostic factors and to incorporate biomarkers such as baseline PNN in order to predict the OS. The receiver operating characteristic curve and the corresponding area under the curve would be constructed to estimate the discrimination power of the nomogram. This tool might assist in the clinical decision-making and patient management. Indeed, patients harboring good prognostic factors with a favorable score could be offered a bladder preservation strategy in case they rejected the surgical option.

Our study has several limitations. First, it was limited by its retrospective nature with potential patient selection biases and missing data. Second, the study period spans a total of 23 years, inevitably resulting in heterogeneity among patients and clinical practice. However, we tried to reduce this bias by including patients treated with a curative dose to the bladder only and the proportion of 2D/3D versus IMRT planification was similar. Third, we did not report specific survival nor the invasive nature of local recurrence but the majority of local failures are reported to be non-muscle-invasive [34]. Fourth, we chose arbitrarily the median NLR and neutrophil as cut-off. Even though there is no consensus on the optimal threshold, the median values are frequently used in the literature [12,16]. The kinetic evolution of biological parameters during and after treatment could not be assessed because of missing data. It could be of interest to analyze the impact of radiation-induced lymphopenia on post-treatment NLR and outcomes. Indeed, irradiation of draining lymph nodes, which represent the main site of T-cell cross-priming by dendritic cells, could affect immune cell functions and migration [41], and could therefore result in lymphopenia. In our study, nearly all patients received elective pelvic lymph node irradiation and approximately half of them were treated with intensity-modulated radiation. Modern techniques such as volumetric modulated arc therapy result in larger volumes of healthy tissues receiving low doses of radiation that could affect circulating lymphocytes [42]. Finally, studying the prognostic role of other peripheral blood markers including platelets and hemoglobin could help the risk stratification in MIBC patients with bladder-sparing treatment.

## 5. Conclusions

Among patients with MIBC treated with CRT, NLR was not associated with survival outcomes. Interestingly, an elevated neutrophil count at baseline (>4000/mm^3^) was associated with worse OS and PFS. Prospective studies are necessary to validate the prognostic and predictive values of this marker. If this is confirmed prospectively, baseline neutrophil count could be an interesting tool for risk stratification in localized MIBC, prediction of survival, and treatment personalization.

## Figures and Tables

**Table 1:** Patients’ characteristics (*n* = 194).

Follow-Up (Months)	
Median [range]	37.5 [1.0–213.5]
**Center, *n* (%)**	
Besançon	111 (57%)
Dijon	83 (43%)
**Gender, *n* (%)**	
Men	144 (74%)
Women	50 (26%)
**Age at diagnosis**	
Mean (SD)	77.3 (7.1)
Median [range]	79.0 [55.0–94.0]
**Charlson score, *n* (%)**	
<5	17 (9%)
≥5	177 (91%)
**Hydronephrosis, *n* (%)**	
No	128 (68%)
Yes	59 (32%)
Missing	7
**Histology, *n* (%)**	
Urothelial carcinoma	181 (93%)
Other	13 (7%)
**CIS associated, *n* (%)**	
No	167 (88%)
Yes	22 (12%)
Missing	5
**T stage, *n* (%)**	
T2	165 (85%)
T3–T4	38 (15%)
Missing	1
**N stage, *n* (%)**	
N0	170 (89%)
N+	22 (11%)
Missing	2
**Tumor size, *n* (%)**	
≤5 cm	69 (57%)
>5 cm	51 (43%)
Missing	74
**Unifocal/Multifocal, *n* (%)**	
Unifocal	122 (69%)
Multifocal	56 (31%)
Missing	16
**Complete TURBT, *n* (%)**	
No	40 (28%)
Yes	102 (72%)
Missing	52
**Neoadjuvant chemotherapy, *n* (%)**	
No	170 (88%)
Yes	24 (12%)
**Kidney failure, *n* (%)**	
No	113 (60%)
Yes	77 (40%)
Missing	4
**CRT choice, *n* (%)**	
Surgery refusal	59 (31%)
Surgical contraindication	129 (69%)
Missing	6
**Baseline neutrophil count, *n* (%)**	
Median [range]	4000.0 [1500.0–16,858.0]
**Baseline lymphocyte count, *n* (%)**	
Median [range]	1625.0 [190.0–3700.0]
**Baseline NLR, *n* (%)**	
Median [range]	2.6 [0.6–19.2]

Abbreviations: SD: standard deviation; CIS: carcinoma in situ; TURBT: transurethral bladder tumor resection; CRT: chemoradiation; NLR: neutrophil-to-lymphocyte ratio.

**Table 2:** Patients’ characteristics according to baseline NLR and baseline neutrophil count (*n* = 178).

	Baseline NLR ≤ 2.6	Baseline NLR > 2.6	*p*-Value	Baseline PNN ≤ 4000	Baseline PNN > 4000	*p*-Value
**Follow-up (months)**			0.23			0.48
Median [range]	41.3 [1.6–213.5]	32.5 [1.5–169.0]		42.4 [1.6–213.5]	36.6 [1.5–169.0]	
**Center, *n* (%)**			0.24			0.53
Besançon	54 (59%)	44 (50.6%)		50 (57.5%)	48 (52.7%)	
Dijon	37 (41%)	43 (49.4%)		37 (42.5%)	43 (47.3%)	
**Gender, *n* (%)**			0.50			**0.04**
Male	65 (72%)	66 (76%)		58 (66.7%)	73 (80.2%)	
Female	26 (28%)	21 (24%)		29 (33.3%)	18 (19.8%)	
**Age at diagnosis**			0.36			0.89
Mean (SD)	77.7 (7.3)	77.2 (6.9)		77.2 (7.5)	77.7 (6.7)	
Median [range]	79.0 [58.0–94.0]	78.0 [55.0–93.0]		79.0 [58.0–94.0]	79.0 [56.0–93.0]	
**Charlson score, *n* (%)**			0.14			0.10
<5	11 (12%)	5 (6%)		11 (13%)	5 (5.5%)	
≥5	80 (88%)	82 (94%)		76 (87%)	86 (94.5%)	
**Hydronephrosis, *n* (%)**			**0.04**			0.19
No	66 (75%)	50 (60%)		61 (73%)	55 (63%)	
Yes	22 (25%)	33 (40%)		23 (27%)	32 (37%)	
Missing	3	4		3	4	
**Histology, *n* (%)**			0.14			0.10
Urothelial carcinoma	83 (91%)	84 (97%)		79 (91%)	88 (97%)	
Other	8 (9%)	3 (3%)		8 (9%)	3 (3%)	
**CIS associated, *n* (%)**			0.59			0.13
No	78 (89%)	73 (86%)		70 (83%)	81 (91%)	
Yes	10 (11%)	12 (14%)		14 (17%)	8 (9%)	
Missing	3	2		3	2	
**T stage, *n* (%)**			0.60			0.49
T2	78 (87%)	73 (84%)		76 (87%)	76 (83.5%)	
T3–T4	12 (13%)	14 (16%)		11 (13%)	15 (16.5%)	
Missing	1	0		1	0	
**N stage, *n* (%)**			0.77			0.19
N0	80 (90%)	77 (89%)		75 (86%)	83 (92%)	
N+	9 (10%)	10 (11%)		12 (14%)	7 (8%)	
Missing	2	0		1	1	
**Tumor size, *n* (%)**			0.38			0.24
≤5 cm	35 (59%)	26 (51%)		33 (61%)	28 (50%)	
>5 cm	24 (41%)	25 (49%)		21 (39%)	28 (50%)	
Missing	32	36		33	35	
**Unifocal/Multifocal, *n* (%)**			0.91			0.37
Unifocal	56 (68%)	54 (67%)		57 (70%)	53 (64%)	
Multifocal	27 (32%)	27 (33%)		24 (30%)	30 (36%)	
Missing	8	6		6	8	
**Complete TURBT, *n* (%)**			0.33			**0.02**
No	18 (26%)	20 (34%)		12 (20%)	26 (39%)	
Yes	51 (74%)	39 (66%)		49 (80%)	41 (61%)	
Missing	22	28		26	24	
**Neoadjuvant chemotherapy, *n* (%)**			0.13			0.20
No	77 (85%)	80 (92%)		74 (85%)	83 (91%)	
Yes	14 (15%)	7 (8%)		13 (15%)	8 (9%)	
**Kidney failure, *n* (%)**			0.69			**0.01**
No	55 (60%)	50 (57%)		60 (69.0%)	45 (49.5%)	
Yes	36 (40%)	37 (43%)		27 (31.0%)	46 (50.5%)	
**CRT choice, *n* (%)**			0.12			0.26
Surgery refusal	25 (28%)	33 (39%)		32 (38%)	26 (29.5%)	
Surgical contraindication	64 (72%)	51 (61%)		53 (62%)	62 (70.5%)	
Missing	2	3		2	3	
**RT technique, *n* (%)**			0.05			0.29
2D/3D	52 (58%)	36 (43%)		40 (47%)	48 (55%)	
IMRT/VMAT	37 (42%)	47 (57%)		45 (53%)	39 (45%)	
Missing	2	4		2	4	
**Local recurrence, *n* (%)**			0.84			**0.01**
No	70 (84%)	59 (83%)		71 (91%)	58 (76%)	
Yes	13 (16%)	12 (17%)		7 (9%)	18 (24%)	
Missing	8	16		9	15	
**Metastatic recurrence, *n* (%)**			0.28			0.19
No	52 (67%)	40 (58%)		52 (67.5%)	40 (57%)	
Yes	26 (33%)	29 (42%)		25 (32.5%)	30 (43%)	
Missing	13	18		10	21	
**Maximum acute toxicity, *n* (%)**			0.74			0.86
G0	13 (14%)	175(18%)		165 (17%)	13 (15%)	
G1–2	72 (79%)	66 (78%)		66 (77%)	79 (80%)	
G3	6 (7%)	4 (5%)		5 (6%)	5 (5%)	
Missing	0	2		1	1	
**Acute hematological toxicity, *n* (%)**			0.09			0.35
G0	61 (68%)	66 (78%)		58 (68%)	69 (77%)	
G1–2	25 (28%)	19 (22%)		24 (28%)	20 (22%)	
G3	4 (4%)	0 (0%)		3 (3%)	1 (1%)	
Missing	1	2		2	1	
**Acute renal toxicity, *n* (%)**			0.91			0.09
G0	82 (90%)	77 (91%)		81 (94%)	78 (87%)	
G1	9 (10%)	8 (9%)		5 (6%)	12 (13%)	
Missing	0	2		1	1	
**Acute urinary toxicity, *n* (%)**			0.51			0.30
G0	35 (39%)	32 (38%)		36 (42%)	31 (34%)	
G1–2	56 (61%)	51 (60%)		50 (58%)	57 (63%)	
G3	0 (0%)	2 (2%)		0 (0%)	2 (2%)	
Missing	0	2		1	1	
**Acute gastrointestinal toxicity, *n* (%)**			0.87			0.87
G0	37 (41%)	38 (45%)		35 (41%)	40 (44%)	
G1–2	52 (57%)	45 (53%)		49 (57%)	48 (54%)	
G3	2 (2%)	2 (2%)		2 (2%)	2 (2%)	
Missing	0	2		1	1	
**Maximum late toxicity, *n* (%)**			0.80			0.90
G0	45 (61%)	44 (63%)		46 (61%)	43 (62%)	
G1–2	29 (39%)	26 (37%)		29 (38%)	26 (38%)	
Missing	17	17		12	22	
**Late urinary toxicity, *n* (%)**			0.78			0.84
G0	47 (63.5%)	46 (66%)		49 (65%)	44 (64%)	
G1–2	27 (36.5%)	24 (34%)		26 (35%)	25 (36%)	
Missing	17	17		12	22	
**Late gastrointestinal toxicity, *n* (%)**			0.91			0.13
G0	67 (90.5%)	63 (90%)		65 (87%)	65 (94%)	
G1	7 (9.5%)	7 (10%)		10 (13%)	4 (6%)	
Missing	17	17		12	22	

Abbreviations: NLR: neutrophil-to-lymphocyte ratio; PNN: neutrophil count; SD: standard deviation; CIS: carcinoma in situ; TURBT: transurethral bladder tumor resection; CRT: chemoradiation; G, grade.

**Table 3:** Overall survival in the univariate and multivariate Cox model.

	HR	95% CI	*p*-Value
**Univariate Cox Model**			
**Age at diagnosis**			0.60
≥80 years vs. <80 years	0.89	[0.58–1.37]	
**CIS associated**			0.21
Yes vs. no	0.59	[0.26–1.35]	
**T**			**0.003**
T3–T4 vs. T2	2.17	[1.30–3.60]	
**N**			0.77
N1–3 vs. N0	0.90	[0.47–1.75]	
**Hydronephrosis**			**0.04**
Yes vs. no	1.60	[1.02–2.50]	
**Complete TURBT**			0.49
Yes vs. no	0.82	[0.47–1.43]	
**Multifocal/unifocal**			0.10
Multifocal vs. unifocal	1.46	[0.93–2.29]	
**Baseline neutrophil count**			**<0.001**
>4000 vs. ≤4000	2.63	[1.68–4.11]	
**Baseline NLR**			**0.02**
>2.6 vs. ≤2.6	1.65	[1.07–2.54]	
**Multivariate Cox analysis with the NLR model (*n* = 123)**			
**T**			0.01
T3–T4 vs. T2	2.49	[1.23–5.03]	
**N**			0.37
N1–3 vs. N0	1.59	[0.57–4.43]	
**Hydronephrosis**			0.65
Yes vs. no	1.15	[0.64–2.06]	
**CIS associated**			0.29
Yes vs. no	0.53	[0.16–1.72]	
**Complete TURBT**			0.09
Yes vs. no	0.58	[0.30–1.09]	
**Baseline NLR**			0.15
>2.6 vs. ≤2.6	1.50	[0.87–2.57]	
**Age at diagnosis**			0.84
≥80 years vs. <80 years	1.06	[0.59–1.90]	
**Multivariate Cox analysis with the neutrophil model (*n* = 123)**			
**T**			0.002
T3–T4 vs. T2	3.12	[1.52–6.41]	
**N**			0.94
N1–3 vs. N0	0.96	[0.33–2.79]	
**Hydronephrosis**			0.93
Yes vs. no	0.98	[0.54–1.76]	
**CIS associated**			0.55
Yes vs. no	0.69	[0.21–2.29]	
**Complete TURBT**			0.02
Yes vs. no	0.47	[0.25–0.90]	
**Baseline neutrophil count**			**<0.001**
>4000 vs. ≤4000	3.32	[1.81–6.06]	
**Age at diagnosis**			0.51
≥80 years vs. <80 years	1.22	[0.69–2.15]	

Abbreviations: HR, hazard ratio; CI, confidence interval; vs.: versus; CIS: carcinoma in situ; TURBT: transurethral bladder tumor resection; NLR: neutrophil-to-lymphocyte ratio.

**Table 4:** Progression-free survival in the univariate and multivariate Cox model.

	HR	95% CI	*p*-Value
**Univariate Cox model**			
**Age at diagnosis**			0.55
≥80 years vs. <80 years	0.90	[0.62–1.29]	
**CIS associated**			0.96
Yes vs. no	0.98	[0.55–1.75]	
**T**			**0.00**
T3–T4 vs. T2	2.08	[1.32–3.29]	
**N**			0.56
N1–3 vs. N0	0.84	[0.46–1.53]	
**Hydronephrosis**			**0.01**
Yes vs. no	1.73	[1.18–2.54]	
**Complete TURBT**			0.83
Yes vs. no	0.95	[0.60–1.50]	
**Multifocal/unifocal**			0.12
Multifocal vs. unifocal	1.36	[0.92–2.01]	
**Baseline neutrophil count**			**<0.001**
>4000 vs. ≤4000	2.00	[1.37–2.92]	
**Baseline NLR**			**0.07**
>2.6 vs. ≤2.6	1.40	[0.97–2.03]	
**Multivariate Cox analysis with the NLR model (*n* = 123)**			
**T**			0.12
T3–T4 vs. T2	1.69	[0.88–3.26]	
**N**			0.31
N1–3 vs. N0	1.61	[0.65–4.02]	
**Hydronephrosis**			0.36
Yes vs. no	1.27	[0.76–2.15]	
**Complete TURBT**			0.35
Yes vs. no	0.78	[0.46–1.32]	
**CIS associated**			0.31
Yes vs. no	1.44	[0.72–2.89]	
**Baseline NLR**			0.52
>2.6 vs. ≤2.6	1.17	[0.73–1.86]	
**Age at diagnosis**			0.96
≥80 years vs. <80 years	0.99	[0.61–1.59]	
**Multivariate Cox analysis with the neutrophil model (*n* = 123)**			
**T**			0.08
T3–T4 vs. T2	1.82	[0.94–3.51]	
**N**			0.54
N1–3 vs. N0	1.22	[0.53–3.38]	
**Hydronephrosis**			0.47
Yes vs. no	1.21	[0.72–2.04]	
**Complete TURBT**			0.19
Yes vs. no	0.70	[0.41–1.19]	
**CIS associated**			0.17
Yes vs. no	1.64	[0.81–3.31]	
**Baseline neutrophil count**			**0.02**
>4000 vs. ≤4000	1.82	[1.12–2.95]	
**Age at diagnosis**			0.82
≥80 years vs. <80 years	1.06	[0.66–1.70]	

Abbreviations: HR, hazard ratio; CI, confidence interval; vs.: versus; CIS: carcinoma in situ; TURBT: transurethral bladder tumor resection; NLR: neutrophil-to-lymphocyte ratio.

## Data Availability

The data presented in this study are available on request from the corresponding author.

## Supplementary Materials

The following supporting information can be downloaded at: https://www.mdpi.com/article/10.3390/cancers15061886/s1, Table S1: Neoadjuvant chemotherapy and chemoradiation characteristics (*n* = 204). Table S2: Acute and late toxicities.
