# Supplementary material for: Elevated Baseline Neutrophil Count Correlates with Worse Outcomes in Patients with Muscle-Invasive Bladder Cancer Treated with Chemoradiation
Source: Cancers (Basel). 2023 Mar 21;15(6):1886. doi: 10.3390/cancers15061886 (PMC10047214; doi:10.3390/cancers15061886)
Supplement: Supplementary file 1 [file cancers-15-01886-s001.zip › cancers-2219328-supplementary/Table S1.pdf]

Table S1. Neoadjuvant chemotherapy and chemoradiation characteristics (n=194)

**Neoadjuvant chemotherapy, *n*(%)**	24 (12%)
Gemcitabine-Cisplatin	8(33.3%)
Methotrexate-Vinblastine-Doxorubicin-Cisplatin	2(8.3%)
Cisplatin-Etoposide	3(12.5%)
Carboplatin-Etoposide	1(4.2%)
Carboplatin-5Fluorouracil	1(4.2%)
Carboplatin-Gemcitabine	1(4.2%)
Unknown chemotherapy	8(33.3%)
**Radiation therapy**	
**Pelvic dose (Gy)**	
Median [range]	45.0 [45.0 - 60.0]
**Bladder dose (Gy)**	
Median [range]	64.8 [50.0 - 70.0]
**Overall treatment time (weeks)**	
Median [range]	7.0 [1.4 - 20.6]
**Interruption, n(%)**	
Yes	21 (11%)
Missing	6
**Technique, n(%)**	
2D/3D	97 (51.6%)
IMRT	91 (48.4%)
Missing	6
**Concurrent chemotherapy**	
**Platinum-based, *n*(%)**	173 (89%)
**Other, *n*(%)**	20 (10%)
**Missing, n(%)**	1 (1%)
**Interruption, *n*(%)**	50 (29%)
Missing	21

Abbreviations: Gy: gray; 2D: two-dimensional ; 3D: three-dimensional ; IMRT: Intensity-modulated radiotherapy
