# Supplementary material for: Elevated Baseline Neutrophil Count Correlates with Worse Outcomes in Patients with Muscle-Invasive Bladder Cancer Treated with Chemoradiation
Source: Cancers (Basel). 2023 Mar 21;15(6):1886. doi: 10.3390/cancers15061886 (PMC10047214; doi:10.3390/cancers15061886)
Supplement: Supplementary file 1 [file cancers-15-01886-s001.zip › cancers-2219328-supplementary/Table S2.pdf]

Table S2. Acute and late toxicities

**Maximum acute toxicity, *n*(%)**	
Grade 0	30 (16%)
Grade 1	113 (60%)
Grade 2	36 (19%)
Grade 3	10 (5%)
Missing	5
**Acute haematological toxicity, *n*(%)**	
Grade 0	138 (74%)
Grade 1	31 (16%)
Grade 2	15 (8%)
Grade 3	4 (2%)
Missing	6
**Acute renal toxicity, *n*(%)**	
Grade 0	170 (90%)
Grade 1	19 (10%)
Missing	5
**Acute cardiac toxicity, *n*(%)**	
Grade 0	184 (97%)
Grade 1	5 (3%)
Missing	5
**Acute urinary toxicity, *n*(%)**	
Grade 0	74 (39%)
Grade 1	94 (50%)
Grade 2	19 (10%)
Grade 3	2 (1%)
Missing	5
**Acute Gastro-intestinal toxicity, *n*(%)**	
Grade 0	84 (45%)
Grade 1	89 (47%)
Grade 2	12 (6%)
Grade 3	4 (2%)
Missing	5
**Maximum late toxicity, *n*(%)**	
Grade 0	98 (63%)
Grade 1	56 (36%)
Grade 2	2 (1%)
Missing	38
**Late urinary toxicity, *n*(%)**	
Grade 0	103 (66%)
Grade 1	51 (33%)
Grade 2	2 (1%)
Missing	38
**Late Gastro-intestinal toxicity, *n*(%)**	
Grade 0	140 (90%)
Grade 1	16 (10%)
Missing	38
